# Ultrasound-Guided Airway Mapping and Regional Blocks in Post-radiation Cervicofacial Contractures: A Case Report

**DOI:** 10.7759/cureus.107808

**Published:** 2026-04-27

**Authors:** Shreyasi Mallick, Rishab Pandita, Deepshikha Patangia, Mithra K

**Affiliations:** 1 Department of Anaesthesiology, Kalinga Institute of Medical Sciences, Kalinga Institute of Industrial Technology (KIIT) University (Deemed to be University), Bhubaneswar, IND

**Keywords:** airway management, airway nerve blocks, awake fibreoptic, contractures, difficult airway, ultrasound

## Abstract

Post-radiation cervicofacial contractures can create major airway management challenges due to anatomical distortion and vascular displacement. We report the case of a 47-year-old woman (American Society of Anesthesiologists (ASA) physical status III), with a history of right hemimandibulectomy and adjuvant radiotherapy for oral squamous cell carcinoma three years prior, presenting for contracture release, with severe trismus (mouth opening <1 fingerbreadth), fixed neck flexion, leftward tracheal deviation, lateralised carotid arteries, and severe malnutrition (BMI 16.2 kg/m²). Preoperative high-frequency ultrasound (6-13 MHz linear probe) enabled the mapping of the trachea, cricoid cartilage, thyroid cartilage, cricothyroid membrane, hyoid bone, bilateral carotid arteries, and internal jugular veins and the identification of displaced critical structures. Under real-time ultrasound guidance, bilateral superior laryngeal nerve blocks and a transtracheal block were performed using a total lignocaine dose of 280 mg (4 mg/kg), avoiding displaced vasculature. Awake nasal fibreoptic intubation with a 7 mm cuffed endotracheal tube was completed uneventfully, after which general anaesthesia proceeded without incident and the patient was extubated at the end of surgery after meeting standard clinical criteria. This case suggests that ultrasound-guided airway blocks, combined with preoperative airway mapping, may facilitate airway topicalisation and help reduce the risk of vascular injury in selected post-radiation patients with distorted anatomy.

## Introduction

Post-radiation cervicofacial contractures following treatment for head and neck cancer are associated with major airway management challenges. Head and neck cancers account for approximately 4-5% of all malignancies worldwide, with over 900,000 new cases diagnosed annually, and the majority of patients receive radiotherapy as primary or adjuvant treatment [1]. Radiation-induced tissue fibrosis develops in up to 30-40% of survivors treated with high-dose cervicofacial radiation, producing progressive trismus, tethered skin, and displacement of vital structures over months to years [1,2]. These changes render standard airway assessment parameters, including Mallampati scoring, thyromental distance, and anatomical landmark palpation, unreliable and substantially increase the risk of failed intubation and unanticipated difficult airway during the induction of general anaesthesia [2]. Point-of-care ultrasound (POCUS) has emerged as a valuable adjunct in this context, enabling the real-time visualisation of displaced structures and guiding regional anaesthesia techniques with precision that surface landmark techniques cannot provide [3,4]. Ultrasound-guided airway blocks, including the superior laryngeal nerve (SLN) block and transtracheal block, permit accurate drug deposition even when surface anatomy is distorted by fibrosis and scarring, thereby improving conditions for awake fibreoptic intubation (AFOI). This report aims to describe the potential utility of ultrasound-guided airway mapping and regional nerve blocks in managing distorted airway anatomy following radical head and neck radiotherapy.

## Case presentation

A 47-year-old woman with a history of right hemimandibulectomy and adjuvant radiotherapy for oral squamous cell carcinoma three years prior presented for cervicofacial contracture release and local flap reconstruction, a shared-airway surgical field with direct proximity to the intubation site, making airway management strategy particularly consequential. She weighed 42 kg (height 161 cm; BMI 16.2 kg/m²), indicating severe protein-energy malnutrition confirmed by serum albumin of 2.6 g/dL. She was classified as American Society of Anesthesiologists (ASA) physical status III on account of malnutrition, chronic anaemia (haemoglobin (Hb) 9.2 g/dL), and restricted cardiopulmonary reserve. Preoperative vitals and laboratory investigations are summarised in Table 1.

**Table 1:** Preoperative baseline vitals and laboratory investigations

Parameter	Patient value	Unit	Reference range
Vital signs			
Heart rate (HR)	84	beats/min	60-100
Blood pressure (BP), systolic/diastolic	118/76	mmHg	<120/<80
Peripheral oxygen saturation (SpO₂) on room air	97	%	≥95
Respiratory rate (RR)	18	breaths/min	12-20
Temperature	37.0	°C	36.5-37.5
Weight	42	kg	-
Height	161	cm	-
Body mass index (BMI)	16.2	kg/m²	18.5-24.9
Haematology			
Haemoglobin (Hb)	9.2	g/dL	12.0-16.0
Haematocrit (Hct)	28	%	36-46
Total leucocyte count (TLC)	7,800	cells/μL	4,000-11,000
Platelet count	220,000	cells/μL	150,000-400,000
Biochemistry			
Serum sodium (Na⁺)	138	mEq/L	136-145
Serum potassium (K⁺)	3.8	mEq/L	3.5-5.0
Serum creatinine	0.7	mg/dL	0.5-1.1
Blood urea nitrogen (BUN)	14	mg/dL	7-20
Random blood glucose (RBG)	102	mg/dL	70-140
Serum albumin	2.6	g/dL	3.5-5.0
Serum total protein	5.4	g/dL	6.4-8.3
Coagulation profile			
Prothrombin time (PT)	13.2	seconds	11-14
International normalised ratio (INR)	1.1	-	<1.2
Activated partial thromboplastin time (aPTT)	28	seconds	25-35
Other investigations			
Electrocardiogram (ECG)	Normal sinus rhythm	-	-
Chest X-ray (CXR)	Clear lung fields; tracheal deviation present	-	-

**Figure 1:**
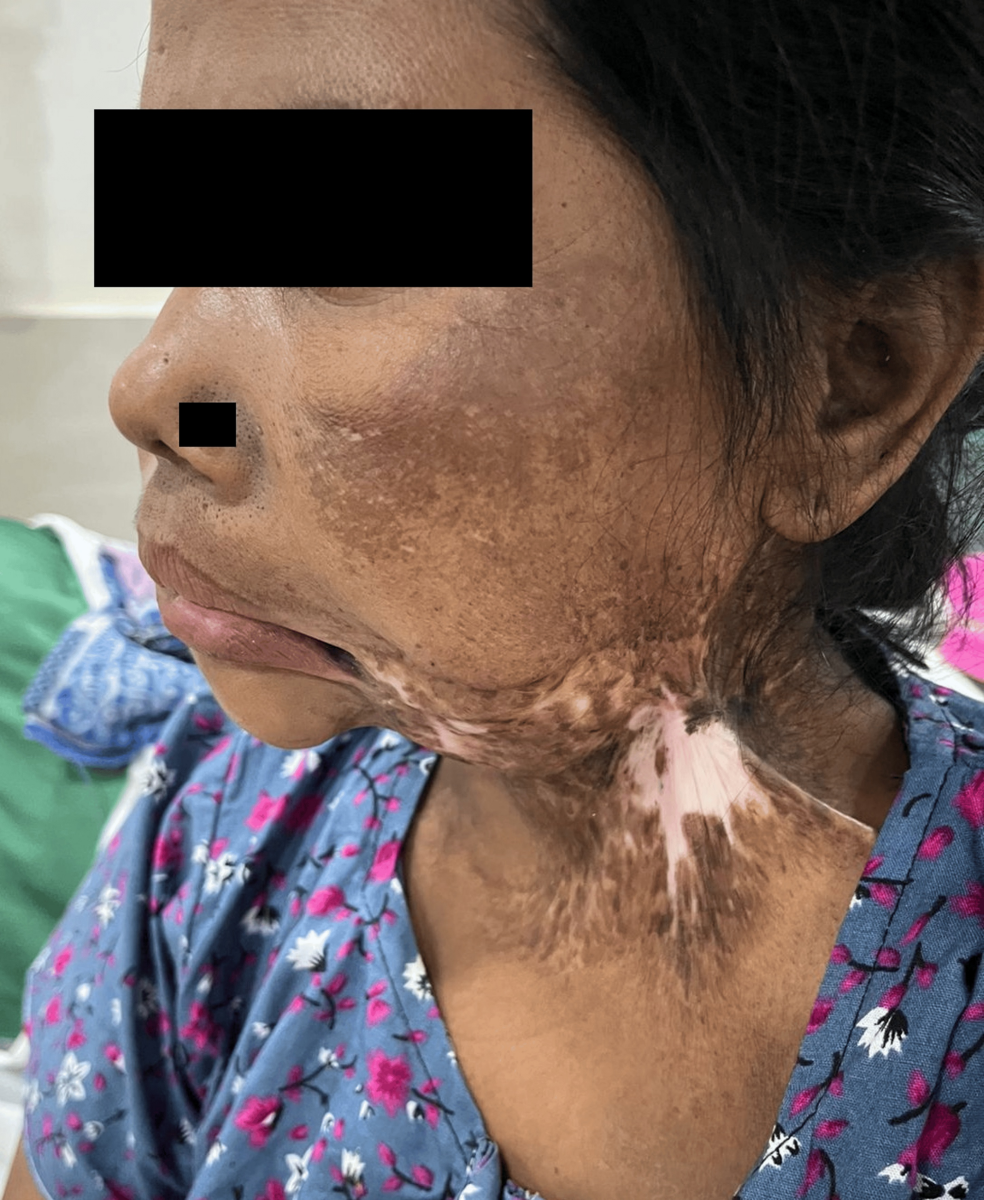
Preoperative clinical photograph demonstrating severe cervicofacial contracture with marked radiation-induced skin changes extending from the left facial region to the anterior neck Note the dense submental and mandibular skin tethering responsible for fixed cervical flexion (approximately 20°) and the restricted mouth opening (inter-incisor distance approximately 10 mm). Facial identifiers including eyebrows, forehead bindi, and distinguishing cloth patterns have been digitally obscured to protect patient identity. Written informed consent was obtained from the patient for publication of this image.

Clinical airway examination revealed severe cervicofacial contracture with marked radiation-induced skin changes extending from the left facial region to the neck (Figure 1). Trismus was profound, with a maximum inter-incisal mouth opening of less than 1 fingerbreadth (approximately 10 mm); therefore, formal Mallampati grading was not possible. Neck extension was nil: the cervical spine was fixed in approximately 20° of flexion due to dense submental and mandibular skin tethering, with an estimated neck extension of 0°. Leftward tracheal deviation was confirmed on inspection and palpation. The fixed neck flexion precluded standard mask positioning. The patient exhibited significant anxiety rooted in a prior traumatic airway experience: a previous anaesthetic attempt following the completion of radiotherapy had been abandoned after failed conventional laryngoscopy, after which AFOI had been attempted without ultrasound guidance. In the absence of targeted regional nerve blocks, topicalisation was inadequate, the patient did not tolerate the procedure, and the attempt was abandoned, leaving the patient with considerable psychological distress surrounding airway instrumentation. Also concerning was the bilateral abnormal lateral displacement of palpable carotid pulsations, raising a serious concern for potential vascular injury during any airway intervention.

**Figure 2:**
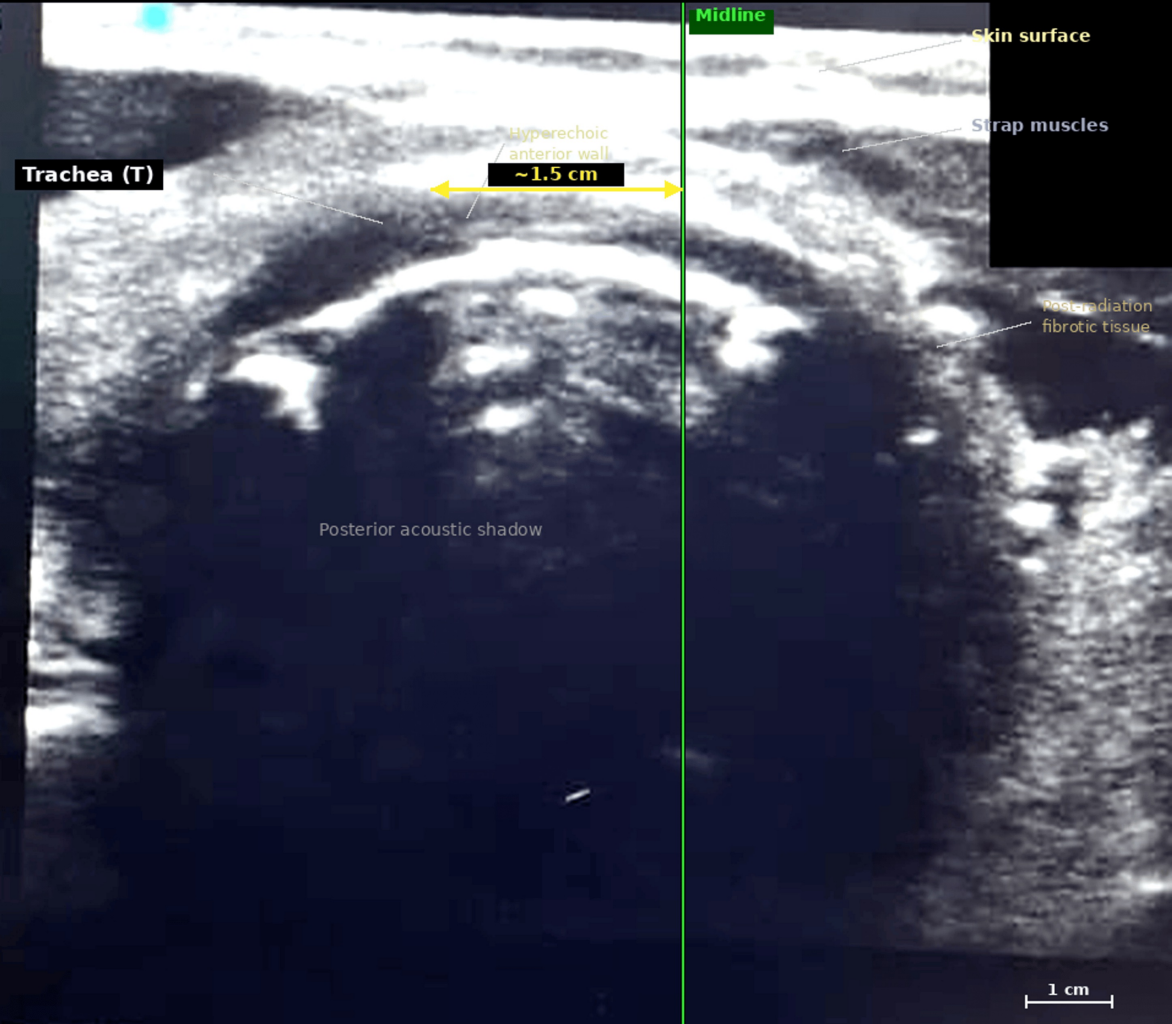
Preoperative transverse neck ultrasonography performed with a high-frequency linear probe (6-13 MHz; SonoSite M-Turbo, Fujifilm, USA) at the level of the cricoid cartilage demonstrating leftward tracheal displacement in the reported patient The trachea (T), identified by its hyperechoic anterior wall arc and characteristic posterior acoustic shadowing from the air-filled lumen, is displaced approximately 1.5 cm to the left of the anatomical midline (green vertical line). The surrounding heterogeneous echogenic tissue reflects post-radiation fibrosis of the cervical soft tissues. The strap muscles (SM) are visible superficially beneath the skin surface (S). Date and time of acquisition have been digitally obscured to protect patient confidentiality.

Recognising the limitations of clinical examination, we employed a high-frequency linear ultrasound probe (6-13 MHz; SonoSite M-Turbo, Fujifilm, USA) with the patient seated upright and neck in neutral position for comprehensive preoperative airway mapping. Systematic scanning in transverse and sagittal planes identified the trachea, cricoid cartilage, thyroid cartilage, cricothyroid membrane, hyoid bone, and bilateral carotid arteries and internal jugular veins [3]. The ultrasound revealed substantial displacement of both the trachea (approximately 1.5 cm to the left of midline) and bilateral carotid vessels from their expected anatomical positions (Figure 2). These identified anatomical structures were marked on the skin surface to guide needle placement and facilitate surgical rescue planning.

A multidisciplinary conference involving anaesthesia, otolaryngology, and surgical teams reviewed the ultrasound findings to formulate a coordinated airway management and rescue plan. The mapped anatomy proved invaluable for planning potential rescue airway sites while identifying vascular structures to avoid during emergency interventions [5]. 

After obtaining informed high-risk consent, ensuring adequate fasting (six hours solid, two hours clear fluids), and confirming baseline investigations, the patient was shifted to the operating room with a fully equipped difficult airway trolley including video laryngoscope, surgical airway set, and fibreoptic bronchoscope. Standard monitoring was applied: 5-lead electrocardiogram (ECG), peripheral oxygen saturation (SpO₂) by pulse oximetry, non-invasive blood pressure (NIBP), and end-tidal carbon dioxide (EtCO₂) capnography via nasal sampling cannula throughout the awake phase. Baseline SpO₂ was 97% on room air. Supplemental oxygen was delivered at 4 L/min via nasal prongs during the entire awake intubation sequence to maintain SpO₂ above 95%. 

Prior to induction, a structured failed airway contingency plan was formulated in accordance with the Difficult Airway Society (DAS) 2015 guidelines for unanticipated difficult intubation, adapted to the awake intubation pathway. The predefined sequential rescue strategy was as follows: in the event of failed AFOI, a first attempt at supraglottic airway device placement (i-gel, size 3) would be made to restore oxygenation. If supraglottic rescue failed, a front-of-neck access via scalpel-bougie-tube cricothyroidotomy would be performed at the pre-marked ultrasound-identified cricothyroid membrane site, taking care to avoid the mapped carotid vessels. Surgical tracheostomy under direct ENT guidance represented the final rescue option. The difficult airway trolley was stocked with a video laryngoscope (C-MAC with D-Blade), i-gel supraglottic airway devices (sizes 3 and 4), a scalpel-bougie-tube emergency front-of-neck access kit, a surgical tracheostomy set, and the fibreoptic bronchoscope. A contoured mask with a two-person technique was pre-planned as the rescue oxygenation strategy. The otolaryngology and head and neck surgical teams were scrubbed and physically present in the operating room throughout the awake intubation phase. Crucially, the cricothyroid membrane had been identified and skin-marked during the preoperative ultrasound mapping session, along with the displaced tracheal midline and carotid vessels, providing a pre-planned safe zone for emergency surgical airway access should it have been required.

Injection glycopyrrolate 0.2 mg intravenously (IV) was administered to reduce airway secretions. Injection midazolam 1 mg IV was titrated for anxiolysis, targeting a Ramsay Sedation Score of 2 (cooperative, oriented, tranquil) to minimise anxiety while preserving spontaneous ventilation and airway reflexes. Oxymetazoline 0.05% nasal drops (two drops per nostril) were instilled bilaterally for mucosal decongestion. Airway topicalisation was achieved with nebulised 4% lignocaine (4 mL=160 mg) over 15 minutes [2]. The total lignocaine dose across all components was 280 mg (4 mg/kg for a 42 kg patient; nebulisation 160 mg+bilateral SLN blocks 2×40 mg+transtracheal block 40 mg), within the maximum safe limit of 7-9 mg/kg for topical and regional airway use. Using real-time ultrasound with a 6-13 MHz linear probe, the SLN block was performed using an out-of-plane approach: a 23-gauge (G) hypodermic needle was guided to deposit 2 mL of 2% lignocaine (40 mg) bilaterally between the greater horn of the hyoid bone and the superior cornu of the thyroid cartilage, just above the thyrohyoid membrane, with vascular colour Doppler confirmation before injection [4,6]. The transtracheal block was performed under direct ultrasound guidance: a 22G IV cannula was advanced through the cricothyroid membrane in the midline, with real-time needle visualisation ensuring the avoidance of the displaced carotid vessels. Free aspiration of air confirmed intratracheal placement, and 2 mL of 2% lignocaine (40 mg) was rapidly injected at end-inspiration to provoke cough and distribute anaesthetic agent across the subglottic mucosa [7]. In standard anatomy, finer-gauge needles (25-27G) are preferred to minimise trauma. A 23G needle was used in our case as its superior echogenicity under ultrasound was judged to reduce the risk of inadvertent vascular puncture more than a finer gauge would reduce the risk of mechanical trauma.

After topicalisation and block onset, AFOI through the right nostril with a 7 mm cuffed flexometallic tube was completed uneventfully, with acceptable patient tolerance and no clinically significant haemodynamic instability. Fibreoptic visualisation revealed a Cormack-Lehane equivalent grade 2 view with adequate glottic exposure achieved on the first pass. The endotracheal tube was railroaded over the bronchoscope without resistance, and tube position was confirmed by visualisation of tracheal rings and carina through the bronchoscope, followed by capnographic waveform confirmation. The total intubation time from bronchoscope insertion to endotracheal tube confirmation was approximately four minutes. Throughout the procedure, the patient remained cooperative and calm with a Ramsay Sedation Score of 2, with no episodes of agitation, coughing, laryngospasm, oxygen desaturation, or haemodynamic instability. General anaesthesia was then induced, and surgery proceeded without complications. Blood loss was minimal, and haemodynamic parameters remained stable throughout the four-hour procedure. The patient was extubated successfully at the end of surgery after meeting standard extubation criteria and demonstrated excellent pain control with multimodal analgesia in the postoperative period.

## Discussion

This case illustrates the practical value of POCUS in the management of anatomically distorted airways. In post-radiation patients, traditional landmark-based techniques for both airway assessment and regional blocks may be unreliable and potentially hazardous because of the unpredictable displacement of vital structures.

Post-radiation fibrosis of the head and neck produces a constellation of airway challenges that evolve over months to years after treatment completion. Radiation damages fibroblasts and vascular endothelium, triggering a chronic inflammatory cascade that culminates in dense fibrosis and contracture [1]. The resultant trismus, reduced cervical mobility, mucosal fragility, and skin tethering not only complicate direct laryngoscopy but also render awake intubation technically demanding by limiting nasal access, narrowing the oropharyngeal inlet, and obscuring supraglottic landmarks. Uzun et al. documented that post-radiation head and neck cancer patients exhibit significantly higher rates of difficult intubation, failed airway, and unplanned surgical airway compared with non-irradiated controls, with the risk compounded by cumulative radiation dose and elapsed time since treatment [1]. Sriramka et al. similarly described the compound challenges of trismus, restricted neck movement, and vascular displacement that characterise post-radiation airways, emphasising that a planned awake technique with robust topicalisation is essential [2].

Ultrasound has transformed airway management in complex cases. Kristensen provided the foundational evidence that high-frequency linear ultrasound reliably identifies the trachea, cricoid cartilage, thyroid cartilage, cricothyroid membrane, and adjacent vascular structures even in patients with distorted anatomy [3]. In post-radiation patients, where palpation-based localisation of these landmarks is unreliable due to fibrosis and anatomical displacement, ultrasound provides real-time imaging guidance that substantially reduces the risk of vascular injury during both regional blocks and emergency surgical airway manoeuvres. Nicholls et al. demonstrated that bedside sonography by emergency physicians significantly reduces time-to-landmark identification for cricothyroidotomy, an advantage that would be amplified in cases with displaced anatomy [5].

The ultrasound-guided regional block technique was particularly crucial in this case. Real-time visualisation ensures the accurate identification of target nerves and avoidance of displaced vessels, significantly reducing the risk of inadvertent vascular puncture or haematoma formation, particularly critical in this patient with laterally displaced carotid arteries. Mohanta et al. demonstrated in a randomised study that ultrasound-guided airway nerve blocks achieved superior intubating conditions compared with nebulisation alone for AFOI, with a lower total lignocaine dose requirement and fewer complications [4]. The SLN block provides sensory anaesthesia to the supraglottic larynx, including the epiglottis, aryepiglottic folds, and false vocal cords, by targeting the internal branch of the SLN as it traverses the thyrohyoid membrane [6]. Shan et al. compared parasagittal and transverse ultrasound-guided approaches to the SLN block and found both to be effective, with ultrasound guidance enabling precise deposition in patients with distorted neck anatomy [6]. The transtracheal block anaesthetises the true vocal cords, subglottis, and upper trachea by producing a cough-reflex mediated spread of local anaesthetic cephalad through the glottis [7]. Together, these three components, namely, nebulisation, bilateral SLN blocks, and a transtracheal block, provide comprehensive topical anaesthesia from the nasopharynx to the upper trachea, creating optimal conditions for AFOI while maintaining spontaneous ventilation [7,8].

Furthermore, the preoperative ultrasound mapping enhanced team preparedness for potential airway emergencies. In the event of failed intubation requiring a surgical airway, the marked tracheal position and documented vascular displacement would have proven invaluable for rapid, safe cricothyroidotomy or tracheostomy [5]. The ASA 2022 Difficult Airway Guidelines explicitly recommend having a pre-formulated rescue strategy, ideally including a marked surgical airway site, in all patients with anticipated difficult airways [8]. Multidisciplinary planning with the otolaryngology and surgical teams, as performed in this case, is consistent with best practice guidelines and is particularly important in post-radiation patients, where the surgical airway itself may be technically challenging.

The success of this approach required careful patient preparation, including detailed counselling about the procedure, judicious sedation to reduce anxiety without compromising airway reflexes, and a team experienced in ultrasound-guided techniques and fibreoptic intubation [8]. The choice of mild sedation (Ramsay Sedation Scale 2) with midazolam rather than deeper sedation was deliberate, given the patient's severe nutritional compromise and the risk of respiratory depression in a contracted airway with limited reserve. Maintaining supplemental nasal oxygen throughout the procedure provided an important safety margin.

Limitations

This is a single case report, and the generalisability of our approach to all post-radiation airway scenarios is limited. The absence of formal preoperative spirometry and quantitative nutritional indices (e.g., prealbumin, subjective global assessment score) represents a reporting gap. Additionally, the fibreoptic view grade during intubation was not formally documented, which would have provided a more objective measure of topicalisation adequacy. Multi-centre case series or prospective registries of ultrasound-guided airway management in post-radiation patients are needed to establish standardised protocols and outcome benchmarks for this high-risk population.

## Conclusions

Preoperative ultrasound airway assessment may represent a valuable adjunct in post-surgical and post-radiation patients with severe cervicofacial contractures, warranting prospective evaluation. Ultrasound-guided regional airway blocks enhance the safety profile of AFOI by enabling precise drug delivery while avoiding displaced vascular structures. This integrated approach, combining advanced imaging, regional anaesthesia, and multidisciplinary collaboration, may help guide clinical decision-making in complex airway scenarios where traditional techniques may prove inadequate or hazardous.

## References

[1] Uzun DD, Zimmermann TN, Schmitt FC (2024). Radiotherapy effects on airway management in patients with nasopharyngeal cancer. Cancers (Basel).

[2] Sriramka B, Karan D, Parida M (2019). Post-radiation airway management - an anesthesiologist's challenge. J Dent Anesth Pain Med.

[3] Kristensen MS (2011). Ultrasonography in the management of the airway. Acta Anaesthesiol Scand.

[4] Mohanta J, Kumar A, Kaushal A, Talawar P, Gupta P, Jain G (2021). Anaesthesia for awake fiberoptic intubation: ultrasound-guided airway nerve block versus ultrasonic nebulisation with lignocaine. Discoveries (Craiova).

[5] Nicholls SE, Sweeney TW, Ferre RM, Strout TD (2008). Bedside sonography by emergency physicians for the rapid identification of landmarks relevant to cricothyrotomy. Am J Emerg Med.

[6] Shan T, Tan Q, Wu D (2024). Ultrasound-guided superior laryngeal nerve block: a randomized comparison between parasagittal and transverse approach. BMC Anesthesiol.

[7] Yadav U, Kumar A, Gupta P (2021). A comparative study of airway nerve blocks and atomized lidocaine by the laryngo-tracheal mucosal atomization device (LMA MADgic) airway for oral awake fiberoptic intubation. Cureus.

[8] Apfelbaum JL, Hagberg CA, Connis RT (2022). 2022 American Society of Anesthesiologists practice guidelines for management of the difficult airway. Anesthesiology.

